# Musical hallucinations – a challenge for psychiatric therapeutical management. Case report

**Published:** 2015

**Authors:** BE Focseneanu, G Marian

**Affiliations:** * ”Titu Maiorescu” University, Faculty of Medicine, Bucharest, Romania; ** ”Prof. Dr. Al. Obregia” Psychiatric Clinical Hospital, Clinic 8 Psychiatry, Bucharest, Romania

**Keywords:** musical hallucinations, hearing loss, obsessive-compulsive disorder

## Abstract

**Background.** Musical hallucinations occur in individuals with and without mental illness, and many patients tend to have intact reality testing. Although literature on musical hallucinations is limited, they have been associated with hearing abnormalities, adverse effects of pharmacological agents, female gender, advances in age and psychiatric illness.

**Aim.** To present the psychiatric management of a case of an old female patient, who suddenly developed verbal and musical hallucinations with a pervasive impact on her daily activities.

**Method.** Female, 71 years old, developed verbal and musical hallucinations 6 months before that have intensified later. She was known with bilateral hypoacusia starting with the age of 45, and magnetic resonance imaging performed 1 year before proved multiple lacunar infarcts. Because of the persistence, most of the time of these auditory hallucinations, the patient experienced pervasive difficulties with her major areas of activities. She was referred to a psychiatric department for evaluation and treatment.

**Results.** The psychiatric consult revealed neither a depressive relapse, nor a mild cognitive impairment, and obsessive-compulsive disorder was suspected with intrusive obsessions. Patient received, as antiobsessional augmentation escitalopram 10mg/ day, an atypical antipsychotic, risperidone, which at 3 mg/ day induced extrapyramidal symptoms and cognitive impairment. Therefore, the dose of risperidone was reduced, extrapyramidal symptoms disappeared, and 300mg/ day of acidum valproicum was initiated.

**Discussion.** Our patient presented with diminished sensory input to the auditory cortex, and it was hard to make a differential diagnosis between an organic and a mental etiology.

**Conclusion.** The integration of musical hallucinations into a psychiatric disorder may be a difficult task, and, their treatment represents a challenge.

## Introduction

Musical hallucinations, labeled as Oliver Sacks syndrome, occur in individuals with and without mental illness, and many patients tend to have intact reality testing. Musical hallucinations represent a specific form of auditory hallucinations whereby patients experience formed songs, instrumental music, or tunes, without an external musical stimulus [**1**-**3**]. Some authors defend that musical hallucinations may be an auditory form of the Charles Bonnet Syndrome (sensory deprivation leads to “release phenomenon” [**4**-**6**] causing an abnormal activity in the otherwise normal segments of cortical music processing modules. Differentiation with subsequent release is proposed to occur due to a decreased cortical auditory input: hearing impairment, structural or chemically modulated disconnection within the auditory pathways, and abnormal excitation of the cortical musical processing modules, among others.

The prevalence of musical hallucinations in a general hospital setting has been estimated to be of 0.16% [**7**], and 2.5% among elderly subjects with hearing complaints [**8**]. Hermesh showed that 26.8% of the outpatient psychiatric patients reported musical hallucinations, while the prevalence rose to 41% among patients with obsessive-compulsive disorder [**9**].

Although literature on musical hallucinations is limited, they have been associated with chronic hearing abnormalities, adverse effects of different pharmacological agents (opioids, tramadol, benzodiazepines, tricyclic antidepressants, carbamazepine, phenytoin, cannabinoids, ketamine, beta-blockers) [**10**], female gender, advanced age, psychiatric illness (depression, schizophrenia, obsessive-compulsive disorder), social isolation, epilepsy [**5**], global atrophy, Hashimoto encephalopathy [**11**,**12**], and focal brain lesions (hemorrhages, metastases, abscesses, arteriovenous malformations, and Listeria rhombencephalitis [**13**,**14**]. The relative importance of each factor remains unclear [**14**]. Many conditions determining hearing loss have been associated with musical hallucinations [**1**,**15**]. Otosclerosis, a condition caused by an abnormal bone homeostasis of the otic capsule that frequently results in hearing impairment in white adults [**16**], has been shown to cause musical hallucinations. The hallucinatory phenomena may arise as a direct consequence of subacute hearing loss caused by otosclerosis, which triggers the auditory cortex [**17**]. 

## Aim

To present the psychiatric management of a case of an old female patient, who suddenly developed verbal and musical hallucinations with pervasive impact on her daily activities.

## Case presentation

Female, 71 years old, widow for 39 years, diagnosed with and treated many years for major depressive disorder, developed 6 months before verbal and musical hallucinations (“talkative voices” and “classical music”) which have intensified later. She was known with bilateral hypoacusia starting with the age of 45, and the MRI performed 1 year before proved multiple lacunar infarcts. Because of the persistence, most of the time of these auditory hallucinations, the patient experienced pervasive difficulties with her major areas of activities, and became very upset. She was referred to a psychiatric department for evaluation and treatment.

At the psychiatric evaluation, the patient was conscious, cooperative, anxious, slightly irritable, with pressurized speech, but coherent. The speech was focused on concentration difficulties in performing domestic tasks and social activities. If initially, she only heard classical music (opera) with mild intensity and pleasant resonance, that comforted her, and prevailed in the evening, the appearance of voices conversing incessantly started to make her tired and began to hinder the daily activities. Gradually she became unable to follow a conversation to an end, to watch a movie, read a book, or look after her 4-year-old niece, although they were not activities that have created problems and they were performed with interest and pleasure. She reported that the frustration level had risen even more as intellectual work performed to increase her revenue become a chore, in the light of severe difficulties with concentration and memory loss. Appetite and sleep were not affected. While young (1978), she had been diagnosed with major depressive disorder for which she was once hospitalized, subsequent episodes being mainly controlled by using tricyclic antidepressants. For about 4 years, the patient was stabilized on a selective serotonin reuptake inhibitor (SSRI) - escitalopram 10mg / day, without side effects. As for the medical background, she was known with bilateral hypoacusia (more accentuated on the left side), peripheral circulatory failure, and hypertension cardiopathy, and treated for many years with diosminum, betahistinum and ginkgo biloba that kept these diseases under control. She was also diagnosed with irritable bowel, whose exacerbations were more pronounced during depressive episodes.

Because the patient’s medical history was enlightening for bilateral hearing loss, she underwent an ENT examination that showed no worsening of the otosclerosis compared to the testing performed two years before and was made available to the patient. The sudden hallucinatory phenomena resulted in a differential diagnosis with lacunar vascular events, but the MRI exam found no additional lesions in the brain than the one conducted a year before. Further neurological examination (including electroencephalogram) excluded the other medical conditions that could have been associated with hallucinations (Parkinson’s disease, vascular epilepsy). Laboratory values were in normal range for blood count, biochemistry, endocrinological and ionogram analysis, except for a slightly higher value of total cholesterol. Excluding organic causes, we launched deeper psychological interviews later, by using psychometric scales for other psychiatric diseases. 

The patient recorded 15 points for Hamilton Depression Raring Scale (HAMD) score, 16 for anxiety at Hamilton Anxiety Scale (HAMA) scale, 29 for Mini-mental state examination (MMSE). The psychiatric consult revealed neither a depressive relapse, nor a mild cognitive impairment, and the obsessive-compulsive disorder was suspected with intrusive obsessions because of their origins from patient’s own head, rather than as ambient sensory perception. Her hallucinations were described as talkative melodious voices and familiar classical music (sometimes famous opera arias sung both instrumentally and vocally) playing repetitively from a radio in her head, without further lateralization. In spite of the amenity music and of songs recognition as heard in her youth and evoking memories from that period, intrusive music playing in her head mostly continuously became annoying and she said she sometimes tried to suppress the musical obsessions by voluntarily singing a different tune in her head or by playing loud music on her TV. She said that intrusive tunes were more frequent and distressing when she was at home, whereas noisy places seemed to make them a little better. On Yale-Brown Obsessive Compulsive Scale (Y-BOCS) she received a total score of 27, which represented severe OC symptoms. An additional diagnose of OCD was made, with good insight.

The patient was treated before with a stable dose of escitalopram 10 mg/ day and lorazepam 1 mg PRN, as a psychiatric treatment. Despite the good insight on the OC symptomatology, the patient received an atypical antipsychotic – risperidone, as an antiobsessional augmentation, starting with 1 mg/ day for 2 weeks, with verbal hallucinations remission and slight improvement of musical hallucinations. The dose of atypical antipsychotic was adjusted at 2 mg/ day with good response after another 2 weeks. At 3 mg/ day, the patient experienced extrapyramidal symptoms, which imposed an association with an anticholinergic agent, with cognitive impairment and worsening of musical hallucinations, instead. Therefore, the dose of risperidone was reduced at the previous level, extrapyramidal symptoms disappeared, and 300mg/ day of acidum valproicum was initiated to cope with mood lability.

Even if musical obsessions were still present, with the “same melody from my early stage”, they became daily transient and felt in the background, with minimal psychological distress (Y-BOCS score: 10).

## Discussion

It has been argued that musical experiences reported by patients with obsessive-compulsive disorder may be phenomenologically distinct from either release phenomena or psychotic hallucinations. Preexisting hearing loss represents a frequent but unnecessary condition in the development of musical hallucinations. Most patients have insight into their condition and perceive the musical hallucinations as intrusive and occasionally unpleasant [**18**]. Our patient presented with diminished sensory input to the auditory cortex, and it was hard to make a differential diagnosis between an organic and a mental etiology. Another observation was that anticholinergic medication use was associated with the worsening of the symptoms because the decrease of the activity in cortical areas involves speech, attention, and memory, especially in elderly patients. Moreover, an elderly patient is more prone to present musical hallucinations and a heightened central nervous system response to inflammatory mediators in the absence of acetylcholine. The connection between the hearing loss and the musical obsessions needs to be evaluated in a further systematical research. Meantime, we have many psychometric tools for the exhaustive evaluation of each patient, and more focus on obsession substrate needs to be considered. 

**Conclusion**

Although musical hallucinations are a rare phenomenon, psychiatrists should try to evaluate their existence more often, as patients are frequently too embarrassed to refer to them freely.

The integration of musical hallucinations into a psychiatric disorder may be a difficult task, and their treatment represents a challenge. Based on the patient’s good insight and compliance, and lack of other neurological evidence, we were able to find the underlying obsession mechanism and to manage this case with good resolution.

**Disclosure**

The authors declare they have no conflict of interest.
